# Wave-of-Advance Models of the Diffusion of the Y Chromosome Haplogroup R1b1b2 in Europe

**DOI:** 10.1371/journal.pone.0021592

**Published:** 2011-06-24

**Authors:** Per Sjödin, Olivier François

**Affiliations:** 1 Department of Evolutionary Biology, Evolutionary Biology Centre, Uppsala University, Norbyvägen, Uppsala, Sweden; 2 University Joseph Fourier Grenoble, Centre National de la Recherche Scientifique, TIMC-IMAG UMR 5525, Mathematical and Computational Biology, Grenoble, France; Institut de Biologia Evolutiva - Universitat Pompeu Fabra, Spain

## Abstract

Whether or not the spread of agriculture in Europe was accompanied by movements of people is a long-standing question in archeology and anthropology, which has been frequently addressed with the help of population genetic data. Estimates on dates of expansion and geographic origins obtained from genetic data are however sensitive to the calibration of mutation rates and to the mathematical models used to perform inference. For instance, recent data on the Y chromosome haplogroup R1b1b2 (M269) have either suggested a Neolithic origin for European paternal lineages or a more ancient Paleolithic origin depending on the calibration of Y-STR mutation rates. Here we examine the date of expansion and the geographic origin of hgR1b1b2 considering two current estimates of mutation rates in a total of fourteen realistic wave-of-advance models. We report that a range expansion dating to the Paleolithic is unlikely to explain the observed geographical distribution of microsatellite diversity, and that whether the data is informative with respect to the spread of agriculture in Europe depends on the mutation rate assumption in a critical way.

## Introduction

Since the development of molecular markers, genetics has been extensively used to address the question of the diffusion of agriculture into Europe, one of the long-standing debates in archaeology and anthropology [1]–[3]. Though archaeologists have considered more sophisticated models of diffusion in the last decade [4], two scenarios are frequently contrasted in the population genetics literature. In 1971, a seminal work by Ammerman and Cavalli-Sforza used radiocarbon dates from Neolithic sites to propose a “wave-of-advance” model of the spread of agriculture in Europe. In this “demic” process, local population growth and migration produce demographic expansion following a traveling wave from the southeast to the northwest of Europe [5]. The wave-of-advance model argues that farmers expanded into Europe from West-Asia about 10,000 years ago, and replaced resident hunter-gatherers with little or no genetic admixture [6], [7]. Alternatively, several archaeologists have hypothesized a cultural model of the development of agriculture, where cultivated plants, domesticated animals and the associated agricultural techniques were adopted with only limited human movements [3], [8]. According to this “cultural” model, the Neolithic farmers did not migrate. Instead the technologies were transmitted to the resident hunter-gatherers who changed their lifestyle and converted to farming. Yet the prehistory of European populations is poorly understood, and the debate between the demic and cultural diffusion models is still active today.

Inference on demographic history of European populations is commonly based on the estimation of coalescent ages of mitochondrial and Y chromosome haplogroups in modern populations [9]–[19]. Estimations of times since the most recent ancestor (TMRCAs) based on mtDNA have suggested a Paleolithic origin of European maternal lineages [10]–[12], but see [20], [21] for suggestions of a Neolithic contribution to the maternal gene pool. In contrast, several studies of Y chromosome haplogroups have suggested more recent origins for the paternal lineages [14], [15], [17], [22], [23]. The results in the latter studies have been interpreted as support for the demic diffusion model, implying that distinct migration patterns took place for women and men in Europe. Offering a more direct view of the past, ancient mtDNA has been recently used to genetically characterize a farming population of the Linear Pottery Culture in Central Europe [24]–[26]. Whereas Haak et al [24] lent weight to the arguments for a Paleolithic origin of Europeans, the subsequent analyses supported little admixture with hunter-gatherer populations [25], genetic affinities of Neolithic farmers with West-Asian populations and significant post-Neolithic events [26].

Among European Y chromosome lineages, haplogroup (hg) R1b1b2 (R-M269) is carried by 110 million European men, and increases in frequency from east to west [27]. Using germline mutation rates (GMR), Balaresque et al [15] reported that the distribution of hgR1b1b2 microsatellite diversity is best explained by spread from a single source in the Near East during the Neolithic. Mutation rate assumptions, however, have a large impact on molecular dating. Using the evolutionary mutation rate (EMR) proposed by Zhivotovsky et al [28], [29], Morelli et al [16] found strong support for considerably older TMRCAs than estimated in [15]. Here we re-investigate whether the spatial distribution of microsatellite diversity of hgR1b1b2 supports a demic or a cultural model of expansion of agriculture into Europe, or if it results from a more recent expansion as suggested in [26]. In previous studies, TMRCAs and population growth rates were estimated using the computer program BATWING, which assumes a model of exponential population growth and divergence without gene flow [30]. The EMR was introduced to correct for the inaccuracy of approximations made by demographic models such as those assumed in BATWING [16]–[18], [29], and it has been observed that the use of corrected rates can increase TMRCA estimates by a 3-fold factor [18]. Another shortcoming of the BATWING model is its failure to reproduce the characteristics of a wave-of-advance, where recurrent founder events occur during range expansion. In addition, estimating the TMRCA of a haplogroup is not necessarily relevant to the study of the expansion time of this haplogroup [3], [20].To overcome these issues and better evaluate the alternative hypotheses for expansion dates, we implemented 14 realistic wave-of-advance models using GMR or EMR estimates as proposed in previous studies [15]–[17]. The wave-of-advance models are designed to capture the history of hgR1b1b2 implicit in the assumptions of cultural or demic diffusion of agriculture in Europe [6], [27] and scenarios of more recent expansions. We also discuss the implications of using GMR estimates in a demic expansion model for the hgR1b1b2 data.

## Results and Discussion

**Figure 1:**
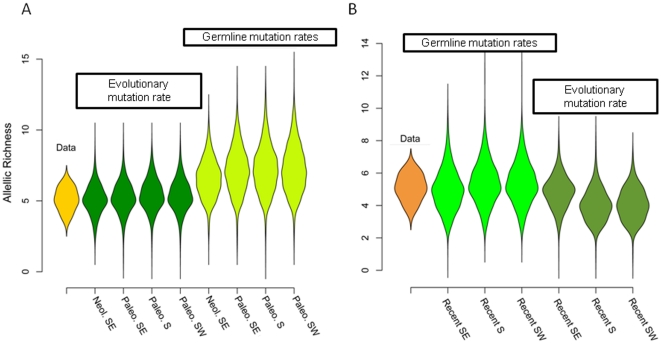
Distributions of allelic richness in 14 range expansion models. Model names refer to the description given in **Table 1**.

We used nine microsatellite markers from 840 European Y-chromosomes typed from hgR1b1b2 (R-M269), a common haplogroup in Europe [15]. Fourteen distinct wave-of-advance models were fitted to the microsatellite allelic richness and to the geographical distribution of microsatellite diversity using computer simulations. The models sorted into three main categories representing the alternative hypotheses of a Paleolithic (21ky ago), Neolithic (10Ky ago) or post-Neolithic (3Ky ago) expansion. Two distinct calibrations of the Y-STR mutation rates, GMR and EMR, were used for each demographic scenario (see Materials and Methods and Table 1 and Figure 1).

**Table 1:** The 14 wave-of-advance models.

	Temporal Origin	Spatial Origin	Mutation Rates	Population Density (ind/km^2^)	Carrying Capacity
**Neol SE–EMR**	10.5 ky BP^1^	South East^3^	Low^6^	0.5	500
**Paleo SE–EMR**	21 k BP^2^	South East^3^	Low^6^	0.05	50
**Paleo S–EMR**	21 k BP^2^	South^4^	Low^6^	0.05	50
**Paleo SW–EMR**	21 k BP^2^	South West^5^	Low^6^	0.05	50
**Neol SE–GMR**	10.5 ky BP^1^	South East^3^	High^7^	0.5	500
**Paleo SE–GMR**	21 k BP^2^	South East^3^	High^7^	0.05	50
**Paleo S–GMR**	21 k BP^2^	South^4^	High^7^	0.05	50
**Paleo SW–GMR**	21 k BP^2^	South West^5^	High^7^	0.05	50
**Recent SE–EMR**	3 ky BP^1^	South East^3^	Low^6^	0.5	500
**Recent S–EMR**	3 k BP^2^	South^4^	Low^6^	0.5	500
**Recent SW–EMR**	3 k BP^2^	South West^5^	Low^6^	0.5	500
**Recent SE–GMR**	3 ky BP^1^	South East^3^	High^7^	0.5	500
**Recent S–GMR**	3 k BP^2^	South^4^	High^7^	0.5	500
**Recent SW–GMR**	3 k BP^2^	South West^5^	High^7^	0.5	500

1 350 generations ago. In these simulations, Europe was colonized in less than 180 generations (SPLATCHE parameters m = 0.45, r = 0.5).

2 700 generations ago. In these simulations, Europe was colonized in less than 180 generations (SPLATCHE parameters m = 0.45, r = 0.5).

3 100 generations ago. In these simulations, Europe was colonized in less than 50 generations (SPLATCHE parameters m = 0.9, r = 1.0).

4 Anatolian origin 39°N, 32°E.

5 Italian origin 41°N, 13°E.

6 Iberian peninsula origin 40°N, 3°E.

7 6.96×10^−4^ per generation – Evolutionary Mutation Rate (EMR, Zhivotovsky et al 2006).

8 6×10^−4^ to 3×10^−3^ per generation – Germline Mutation Rates (GMR, Balaresque et al 2010).

### Distribution of allelic richness

In the original data the number of alleles observed at each locus ranged between 4 and 6. To test whether similar levels of allelic richness could be reproduced by the 14 wave-of-advance models, genetic variation was simulated at 1,000 microsatellite loci under each model (Figure 1). The levels of allelic richness observed in a large proportion of simulated data sets were compatible with those observed in the original data. However we found significant differences between the 14 simulated distributions of allelic richness (Kruskall-Wallis test *P*<10^−6^). Generally, models based on the EMR estimate provided a better fit to the data than models using GMR estimates. The fit was better regardless of whether a post-Neolithic, a Neolithic or a Paleolithic origin of hgR1b1b2 was assumed. Zhivotovsky et al [29] suggested that the high microsatellite mutation rates estimated from germlines are a consequence of additional genetic drift due to population bottlenecks not taken into account by the simplistic evolutionary models used to estimate mutation rates. While wave-of-advance models incorporate effects of recurrent bottlenecks, these models are still obvious simplifications of human demographic expansions. Our results show that the EMR correction proposed by Zhivotovsky et al appears to be useful in the wave-of-advance simulation framework.

### Spatial distribution of microsatellite genetic diversity

**Figure 2:**
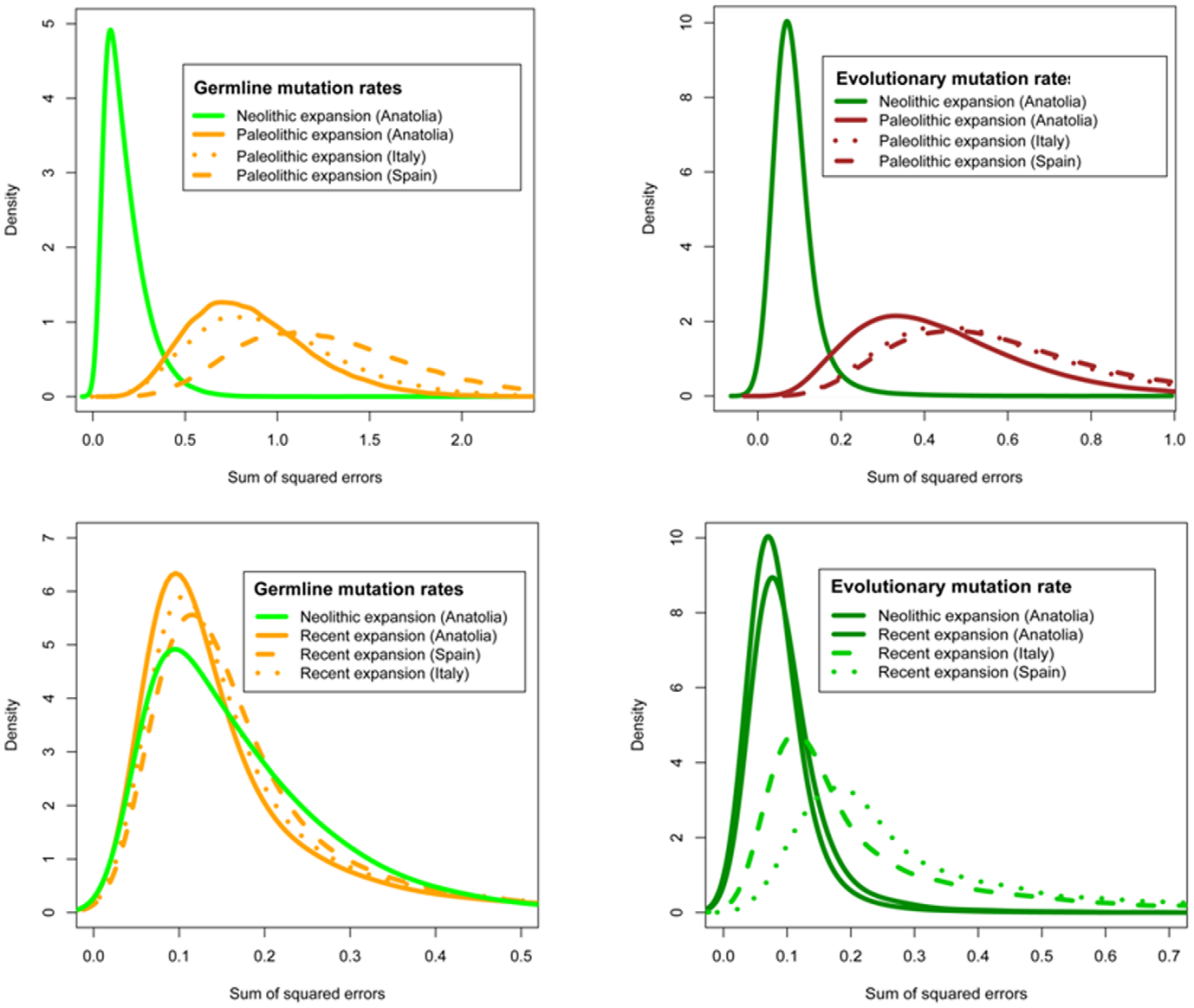
Distribution of sum of squared distances between simulated and observed local microsatellite diversity in 14 range expansion models.

**Figure 3:**
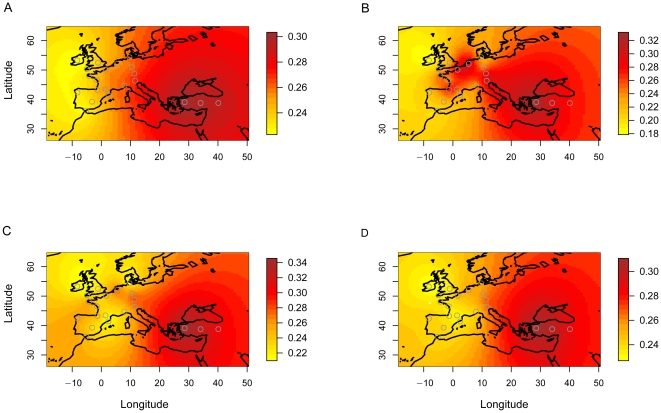
Interpolated maps of sample microsatellite genetic diversity. Best fitting simulation obtained under Model A) Recent expansion from Anatolia (GMR), B) Neolithic expansion from Anatolia (GMR), C) Neolithic expansion from Anatolia (EMR), D) Genetic diversity in the actual data. Circles indicate sample locations.

To investigate if wave-of-advance models could explain the geographic distribution of microsatellite diversity, we restricted our study to simulations that reproduced the number of alleles at each of the 9 microsatellite loci exactly. Using rejection sampling, we produced 100,000 data sets from each model, and measured genetic diversity by the variance in allele size for the 21 population samples in the actual and simulated data sets. Then we evaluated the respective fit of the models by computing the sum of squared differences between the simulated and the actual genetic diversity estimates [31], [32]. Significantly distinct results were produced by the models (*P*<10^−15^). Our results show that an expansion in Neolithic or Mesolithic times (350 generations ago or 10 ky) leads to a lower sum of squared errors than post-glacial re-expansion started 700 generations ago (21 ky ago), regardless of assuming a GMR or EMR model (Figure 2 and Table 2). Using GMRs, simulations of recent (100 generations ago) and rapid expansions from three distinct origins provided a better fit to the geographical distribution of microsatellite diversity than did models with expansion started 350 generations ago. Although models of recent origins using GMRs provided poorer fit than a model of Neolithic expansion using the EMR (Figure 2 and Table 2), the small observed difference makes them however difficult to discriminate (odd ratio = 1.7; Figure 3).

**Table 2:** Sum of squared errors statistics computed over 100,000 replicates of each model.

	Paleolithic Expansion		Neolithic Expansion		Recent Expansion	
	GMR	EMR	GMR	EMR	GMR	EMR
**Mean**	0.858	0.443	0.174	**0.087** *	0.123	0.101
**Median**	0.816	0.408	0.144	**0.075**	0.096	0.085
**SD**	0.332	0.209	0.112	0.050	0.084	0.066

*significant at P<0.0001; All expansions started from Anatolia. GMR: Germline Mutation Rate, EMR: Evolutionary Mutation Rate.

### Comparison to other studies

Using the program BATWING with GMRs, Balaresque et al [15] argued in favor of a Neolithic expansion hypothesis for hgR1b1b2 based on estimates of TMRCAs between 5.5 and 8.0 ky BP. In contrast Morelli et al [16] implemented the EMR and obtained much older TMRCAs, between 14.8 and 32.6 ky BP, supporting a Paleolithic origin. On the other hand, two additional studies employing the EMR obtained TMRCA estimates suggesting that a Neolithic expansion of hgR1b1b2 is more plausible than a Paleolithic expansion [17], [18] (see Supplementary Materials of [18]). Myres et al [17] coalescent estimate for the Y-STR R1b1b2 network tree is 10,270±1,680 years BP, close to the median TMRCAs (8.6–12.2 ky) of the M269 clade obtained by Shi et al [18]. Cruciani et al [33] reported expansion time estimates for hg R1b1b2g and R1b1b2h equal to 8.3 ky BP (95% CI 5.8–10.9ky BP) and 7.4 ky BP (95% CI 5.3–10.2 ky BP) respectively. Their study employed a mutation rate intermediate between the EMR and GMR, and reported TMRCA estimates in-between the estimates of Morelli et al [16] and Balaresque et al [15]. This is in line with Shi et al [18] where the authors investigated the effect of assuming different sets of mutation rates on the outputs of the BATWING algorithm and found that TMRCA estimates based on the EMR are generally larger than estimates based on GMRs. The observations of [18] also imply that if the GMR estimates are the correct rates to use in a spatially expanding population model, some of the previously cited studies would point out to dates of expansion of hgR1b1b2 much more recent than 10,000 years. Moreover, studies of Y-chromosomal haplogroup J, a major haplogroup in south-eastern Europe, have suggested that the importance of more recent expansion events may have been underestimated [34]. Although the result needs confirmation, post-Neolithic expansions are also supported by ancient DNA [26]. In fact the most common Y chromosome hgs in modern Europe are not observed in a population of the earliest farming culture in Central Europe (3 males, [26]). When we used GMR estimates, our results pointed to a similar conclusion. Wave-of-advance models with a recent expansion date received higher support than models of Neolithic expansion (Table 2, Figures 2–3).

### Conclusion

Drawing reliable conclusions about the timing and geographic origin of expansions from genetic data requires a precise modeling of the hypothetic processes that generated the observed genetic variation. As shown previously, estimates of TMRCA are strongly sensitive to prior information on mutation rates [16], [18]. We found that wave-of-advance models can reproduce the geographical distribution of the microsatellite diversity of hg R1b1b2 very accurately (Figure 3). To what extent this distribution supports the demic or cultural dispersal model of agriculture in Europe critically depends on whether the faster germ-line mutation rates or the slower evolutionary mutation rate better capture reality in these models. We found it difficult to discriminate among models assuming EMR (Neolithic expansion) and models assuming GMRs (Recent expansion). An interpretation of our results is as support for the use of the correction proposed by Zhivotovsky et al [28], [29] in wave-of-advance models. Historical events consistent with recent expansions from the south of Europe during the Bronze age [19] or the Greek and Roman civilization in Europe and West Asia [35] cannot be excluded, but the impact of such demographic events on European genomes requires confirmation from ancient DNA studies.

## Materials and Methods

### Data

We used nine microsatellite markers from 840 European Y-chromosomes typed from hgR1b1b2 (R-M269), a common haplogroup in Europe. The population samples were all included in the analyses of Balaresque et al [15]. The data set contained 21 samples from 5 populations from France, 4 from Spain, 3 from the British Isles and Turkey, and 2 from Germany, Italy, Denmark and the Netherlands.

### Wave-of-advance simulations

Simulations of “wave-of-advance” models were performed with the computer program SPLATCHE2 [36]. The program was used to run non-equilibrium stepping-stone simulations on a lattice of demes mirroring the geography of Europe. More specifically, range expansions occurred in a 64×42 lattice of 2,688 demes covering Europe from latitude 38°N to 65°N and from longitude −10°E to 40°E [37]. In the stepping-stone simulations, local populations sent migrants to their nearest neighbors at rate *m*. The establishment probabilities of incoming individuals were inversely proportional to specific friction values that accounted for geographic obstacles, such as mountain areas and seas. Within each deme, the population size grew according to a logistic model with growth rate *r,* and saturated at the carrying capacity, *K*. Based on anthropological data, we used different estimates of population density for expansions started during the late Upper Paleolithic or during the Neolithic. Population density was equal to ∼0.05 individual per km^2^ for Paleolithic populations, and a 10-fold higher value was chosen for farming populations during the Neolithic [6], [37], [38]. To match these prehistoric population density values, carrying capacities were set to *K* = 50 (Paleolithic expansion) and *K* = 500 (Neolithic expansion) in each deme. After the completion of a demographic phase generating a wave-of-advance of populations in Europe, SPLATCHE2 simulates multilocus microsatellite genotypes according to a stepwise mutation model. We simulated nine microsatellite loci for each of the 840 European individuals located at the geographic sites specified in Balaresque et al [15].

### The 14 models

Fourteen distinct wave-of-advance models summarized in Table 1 and Figure 1 were compared to the data using computer simulations. The models sorted into three main categories representing the two alternative hypotheses of a demic or a cultural diffusion of agriculture and a third hypothesis of a more recent expansion scenario [26], [34]. For the demic diffusion model, we assumed that range expansion started around 10ky ago, and the origin of the spread was in Anatolia (39°N, 32°E), southeast to Europe. For the cultural diffusion model, we assumed that range expansion started around 21ky ago, and three distinct origins were considered. Geographic origins in Anatolia, Iberian Peninsula (Spain 40°N, 3°E, southwestern Europe) and Italy (41°N, 13°E, southern Europe) were chosen to mirror the locations of glacial refugia in southern Europe. Recent expansions were started 3Ky ago, and we used the same 3 geographic origins as in the previous models. The SPLATCHE parameters that reproduce these demographic expansion scenarios are given in Table 1. Additionally two Y-STR mutation rate calibrations were included in the models: the comparatively high microsatellite germline mutation rate (GMR) values ranging between 6×10^−4^ and 3×10^−3^ mutation per generation [15] and the lower “evolutionary” mutation rate (EMR, [28]). In the Zhivotovsky method, the ages of haplogroups in populations are estimated using an evolutionary effective mutation rate of Y-STR of 6.96×10^−4^ per generation. In preliminary runs, we also investigated expansions corresponding to the initial colonization of Europe by modern humans around 40,000 years ago (1,500 generations ago) but we did not retain these models due to their poor fit to the observed data.
